# Progressive optic nerve changes in cavitary optic disc anomaly: integration of copy number alteration and cis-expression quantitative trait loci to assess disease etiology

**DOI:** 10.1186/s12881-019-0800-4

**Published:** 2019-04-27

**Authors:** Eileen S. Hwang, Denise J. Morgan, Katie L. Pennington, Leah A. Owen, John H. Fingert, Paul S. Bernstein, Margaret M. DeAngelis

**Affiliations:** 1 Department of Ophthalmology and Visual Sciences, John A. Moran Eye Center, University of Utah School of Medicine, 65 Mario Capecchi Drive, Salt Lake City, UT 84132 USA; 2 Department of Ophthalmology and Visual Sciences, Carver College of Medicine, University of Iowa, Iowa City, IA USA; 3 Department of Pharmacotherapy, College of Pharmacy, University of Utah, Salt Lake City, UT USA; 4 Department of Population Health Sciences, University of Utah School of Medicine, Salt Lake City, UT USA

## Abstract

**Background:**

We performed clinical and genetic characterization of a family with cavitary optic disc anomaly (CODA), an autosomal dominant condition that causes vision loss due to adult-onset maculopathy in the majority of cases. CODA is characterized by a variably excavated optic nerve appearance such as morning glory, optic pit, atypical coloboma, and severe optic nerve cupping.

**Methods:**

Four affected and fourteen unaffected family members of a multi-generation pedigree were phenotyped by visual acuity, intraocular pressure, dilated fundus examination, fundus photography, and optical coherence tomography. Genetic analysis was performed by breakpoint polymerase chain reaction (PCR), long range PCR, and direct Sanger sequencing. The functional relevance of the copy number alteration region was assessed by in silico analysis.

**Results:**

We found progressive optic nerve cupping in three affected members of a family with CODA. In one individual, an optic pit developed over time from a normal optic nerve. By two independent methods, we detected a previously described intergenic triplication that segregated with disease in all adults of the family. The copy number alteration was also detected in five children with normal optic nerves. eQTL analysis demonstrated that this CNA region regulates expression of up to 4 genes in cis.

**Conclusions:**

Morning glory, optic pit and atypical coloboma are currently considered congenital anomalies of the optic nerve, but our data indicate that in CODA, the excavated optic nerve appearance may develop after birth and into adulthood. In silico analysis of the CNA, may explain why vairable expressivity is observed in CODA.

**Electronic supplementary material:**

The online version of this article (10.1186/s12881-019-0800-4) contains supplementary material, which is available to authorized users.

## Background

The optic nerve plays an essential role in vision by connecting the retina, which senses light, to the brain. The innermost part of the optic nerve is called the optic disc. The term cavitary optic disc anomaly (CODA) (OMIM #611543) was first used to describe an autosomal dominant pedigree with a range of optic disc abnormalities including optic pit, atypical optic nerve coloboma, and morning glory optic nerve [1]. The terms atypical optic nerve coloboma and optic pit have also been used to describe autosomal dominant pedigrees with a CODA phenotype [2, 3]. Although CODA-associated optic disc abnormalities are thought to be congenital, vision is typically normal early in life. In approximately half of CODA cases, vision loss occurs in adulthood due to fluid within the macula, the part of the retina responsible for central, sharp vision. The development of macular fluid in CODA is similar to optic pit maculopathy and can be treated with vitrectomy, laser and gas [4]. However, visual recovery can be slow and incomplete [5, 6].

The CODA phenotype in an Iowa family segregated with an intergenic copy number alteration (CNA) on chromosome 12. This intergenic triplication was correlated with expression of a flanking gene, *MMP19,* encoding matrix metalloproteinase 19, in a reporter assay [7]. In this manuscript, the CODA phenotype and its relation to the genotype are described in a four-generation Utah family. We further characterize the CNA and provide evidence that the CODA optic disc phenotype can be adult-onset rather than congenital. Additionally, in silico analysis suggests that this CNA region regulates expression of up to four genes through *cis*-expression quantitative trait loci (cis-EQTLs), which may explain variable expressivity in this disease.

## Methods

The study was prospectively approved by the University of Utah Institutional Review Board (IRB #52879) for collection of genetic and clinical data, and was performed in accordance with the Declaration of Helsinki. Written informed consent for research and publication was obtained from all participants or the parents of participants who were minors. Sixteen family members were examined and extensively characterized by the authors (ESH, PSB). Outside documentation of normal visual acuity and funduscopic examination was reviewed for individuals III.9 and IV.1. Fundus photographs including stereoscopic optic nerve photos were obtained. Spectral domain optical coherence tomography (OCT) images of the optic nerve, retinal nerve fiber layer, and macula were obtained with a Spectralis HRA + OCT (Heidelberg Engineering, Heidelberg, Germany). Individuals were determined to be affected if they had 1) a deep cup with thin rim or 2) a focal grey, white, or yellow excavation in the optic nerve head. Through interview with living family members, we identified two deceased individuals who had bilateral vision loss in their 40s and marked these individuals as presumably affected on the pedigree. All clinical characterization was masked because it was performed prior to genetic analysis. For affected family members, clinical history was determined from medical records review. The presence of macular fluid in affected members was determined by OCT and treated according to standard clinical practice with pars plana vitrectomy, laser, and gas.

Genotyping was performed on leukocyte deoxyribonucleic acid (DNA) that was purified using QIAamp DNA blood Midi Kit (Qiagen, Hilden, Germany). Polymerase chain reaction (PCR) primers were designed to encompass the breakpoint of the CNA using the Primer Blast program [7, 8]. The final primers used for breakpoint PCR were 5′-TCA GGC ACC CCA TGT TCT TT-3’and 5′-TGG CTG TGA GAA CTA TAG GAG G-3′. Breakpoint PCR was performed using a standard protocol. Sanger sequencing was performed at the DNA Sequencing Core Facility, University of Utah.

Sanger sequencing results were aligned to the native sequence using Ensembl genome browser release 92 and the UCSC genome browser human assembly GRCh37/hg19 to identify upstream and downstream breakpoints [9–12]. Long range PCR was performed using the Long Amp Taq PCR kit (New England Biolabs, Ipswich, MA) according to the manufacturer’s instructions with the primers 5′-CAG CGG GCT AAT ACT ACA AGA CTT C-3′ and 5′-CCA GAT GGC TGT GAG AAC TAT AGG A-3′ with a single annealing and extension step of 18 min at 65 °C with 0.5 M betaine for 30 cycles. A QIAquick gel extraction kit (Qiagen, Hilden, Germany) was used to extract the DNA from the gel for sequencing according to the manufacturer’s instructions.

Quantitative PCR was performed using predesigned TaqMan copy number assays (ThermoFisher Scientific, Waltham, Massachusetts, USA). Hs07018987_cn and Hs07018510_cn targeting hg19 chr12:56,241,453 and chr12:56,244,285. RNaseP was used as a reference assay (ThermoFisher Scientific, Waltham, Massachusetts, USA). 10 μl reactions were performed in triplicate using TaqMan Gene Expression MasterMix with 20 ng DNA on an Applied Biosystems 7500 Real Time PCR machine with the reaction mixture and cycle as recommended by the manufacturer (ThermoFisher Scientific, Waltham, Massachusetts, USA). Results were analyzed using CopyCaller v2.1 software (ThermoFisher Scientific, Waltham, Massachusetts, USA).

In silico analyses were performed using the freely available data on GTEx portal version 7, the NCBI gene database, and dbSNP [13–16].

## Results

### Family description

**Fig. 1:**
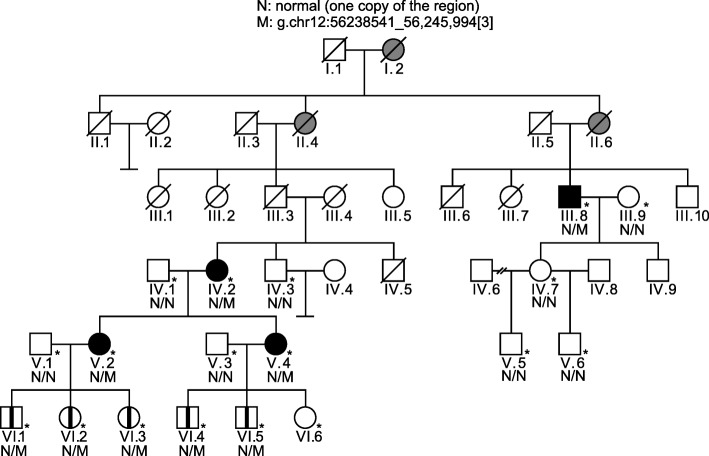
Pedigree of family with cavitary optic disc anomaly. Clinically affected individuals are represented by black symbols, presumably affected individuals are represented by gray symbols, unaffected individuals are represented by open symbols, and asymptomatic carriers are represented by an open symbol with a vertical black line. Examined individuals are marked with asterisks (*). Alleles are denoted by N: normal (one copy), and M: hg19 g.chr12:56238541_56,245,994 [3] (three copies of the region). Through interview with living family members, we identified two deceased individuals who had bilateral vision loss in their 40s and marked these individuals as presumably affected on the pedigree

We identified a family with four living individuals affected by CODA and fourteen living unaffected individuals from four generations (Fig. 1). The founding members of the pedigree (I.1 and I.2) migrated to Utah from Yugoslavia and are deceased. Individuals in the sixth generation ranged in age from 4 years old (VI.6) to 17 years old (VI.1).

### Clinical characterization of affected individuals

**Fig. 2:**
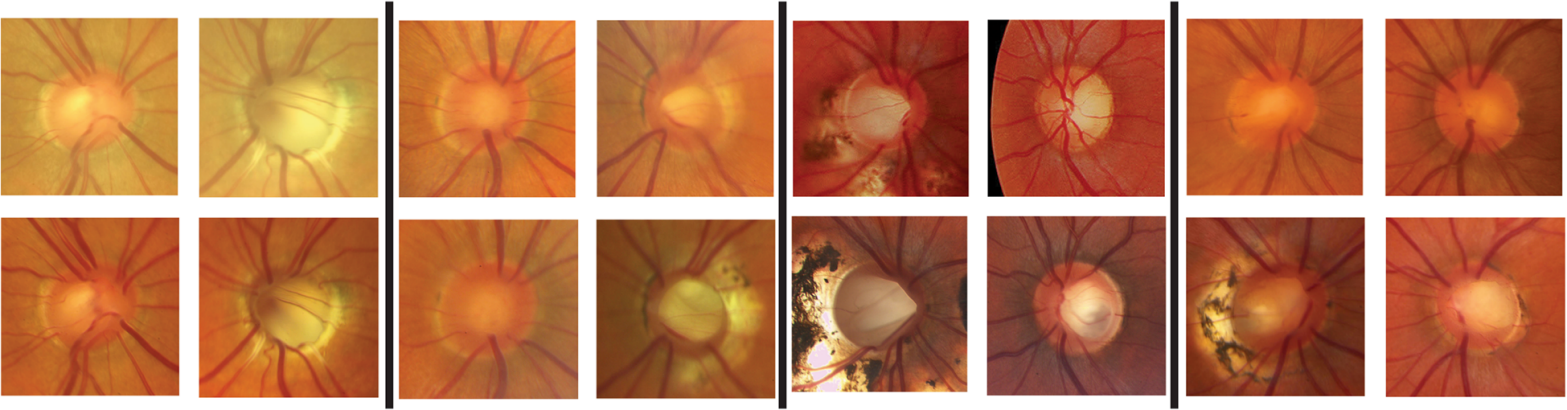
Optic nerve photographs of individuals affected by cavitary optic disc anomaly. Each group of four images (separated by black bars) represents one affected individual. In each group of four, the right eye images are displayed in the first column, left eye images are displayed in the second column, baseline images are displayed in the top row, and follow up images are displayed in the bottom row. Left, individual III.8 at baseline and at 3 year follow up. Second from left, individual IV.2 at baseline and at 2 year follow up. Third from left, individual V.2 at baseline and at 9 year follow up. Right, individual IV.4 at baseline and at 9 year follow up

Affected individuals were followed longitudinally by clinical examination using ophthalmoscopy, fundus photography, and optical coherence tomography (OCT) (Table 1). OCT provides cross-sectional images of the optic disc and macula. Length of follow up ranged from 3 to 14 years. Three of the four affected individuals demonstrated progressive enlargement of the optic cup, the depression within the optic disc (Fig. 2).

**Table 1:** Clinical histories of affected individuals in cavitary optic disc anomaly pedigree

Affected individual	Best corrected visual acuity at presentation		Age at macular fluid onset		Number of treatments		Length of follow up (years)	Best corrected visual acuity at last visit		Macular fluid at last visit	
	right	left	right	left	right	left		right	left	right	left
III.8	20/20	20/250	n/a	27	0	0	5	20/20	20/250	absent	absent
IV.2	20/20	20/70	60	58	0	1	3	20/30	20/25	present	absent
V.2	20/100	20/20	23	35	4	4	14	20/200	20/100	absent	absent
V.4	count fingers	20/20	25	34	1	1	10	20/800	20/125	present	present

Affected individual III.8 reported losing vision in the left eye at age 27, 42 years prior to establishing care at our institution. He saw an ophthalmologist at that time, but no treatment was offered. On presentation at our institution, his right optic nerve had a deep, enlarged cup. In the left eye, his optic cup was abnormally deep, and his retina was atrophic in the temporal macula and mid-periphery. His optic nerve and macula appearance remained stable over a five-year period.

Affected individual IV.2 presented with a central, deep pit in the right eye and a temporal optic pit with macular fluid in the left eye at age 58. The left eye was treated with vitrectomy, laser, and gas. The optic cup enlarged in both the untreated right eye and the treated left eye over a two-year period. After two years, the right eye developed a small amount of fluid adjacent to the optic disc, which was observed because it did not threaten central vision.

At presentation, affected individual V.2 had already had vitrectomy and endolaser of the right eye at an outside institution for macular fluid associated with an optic pit. Her left optic nerve was completely normal. Over the next few years, she was treated multiple times for persistent macular fluid in the right eye, which eventually resolved. During that time period, her left eye remained normal and did not require treatment. She was then lost to follow up until she returned nine years after her first visit with vision loss in the left eye. At that time, the left eye demonstrated a distinct optic pit as well as macular fluid. The left eye was treated was vitrectomy, laser, and gas, and the macular fluid improved dramatically, but recurred four times over the next two years. Each time, she responded to treatment with laser and/or gas.

Affected individual V.4 demonstrated central optic pits in both eyes with small optic cups on presentation. The right eye had macular fluid and underwent vitrectomy, laser, and gas. The left eye had no sight threatening complications at this time. The patient was lost to follow up and returned nine years later with vision loss in the left eye due to macular fluid. Her right eye still had macular fluid but was not treated due to poor prognosis. The left eye underwent vitrectomy, laser, and gas and the macular fluid improved substantially. Over the nine-year period, both optic cups enlarged dramatically.

All affected individuals had normal intraocular pressure (less than 24 mmHg) at all non-post-operative measurements. Visual fields for affected individuals demonstrated defects corresponding to areas of current or past macular fluid (data not shown). Optic nerve OCTs demonstrated very deep cups in the affected eyes (Additional file 1: Figure S1). When the bottom of the cup could be observed on OCT, there was often one area that was deeper than the remainder of the cup, supporting an optic pit phenotype rather than glaucomatous cupping.

### Detection of a chromosome 12 CNA

#### Breakpoint polymerase chain reaction (PCR) assay

Initially, we tested our CODA pedigree for the CNA previously described in another pedigree as a triplication of (hg19) chr12:g.56,238,827-56,244,961 [7]. We detected a CNA, but found that the actual boundaries of the triplicated region are (hg19) chr12:56,238,541–4 and chr12:56,245,994–7, for a length of 7453 bp. Therefore, we designed a breakpoint PCR assay to span the end of one copy and the beginning of the next copy in a tandem repeat and to produce no product with a normal genotype. We initially targeted the previously described breakpoints, but the product we obtained was not of the expected size or sequence. By sequentially designing PCR assays and sequencing the products, we were able to precisely establish the boundaries of the replicated region. With our final breakpoint PCR design, we tested individual III.8 and found a 1.1 kb product, and established that the CNA is tandem and non-inverted. We sequenced the 1.1 kb product and found that the upstream and downstream breakpoints were homologous and contained Alu sequences.

**Fig. 3:**
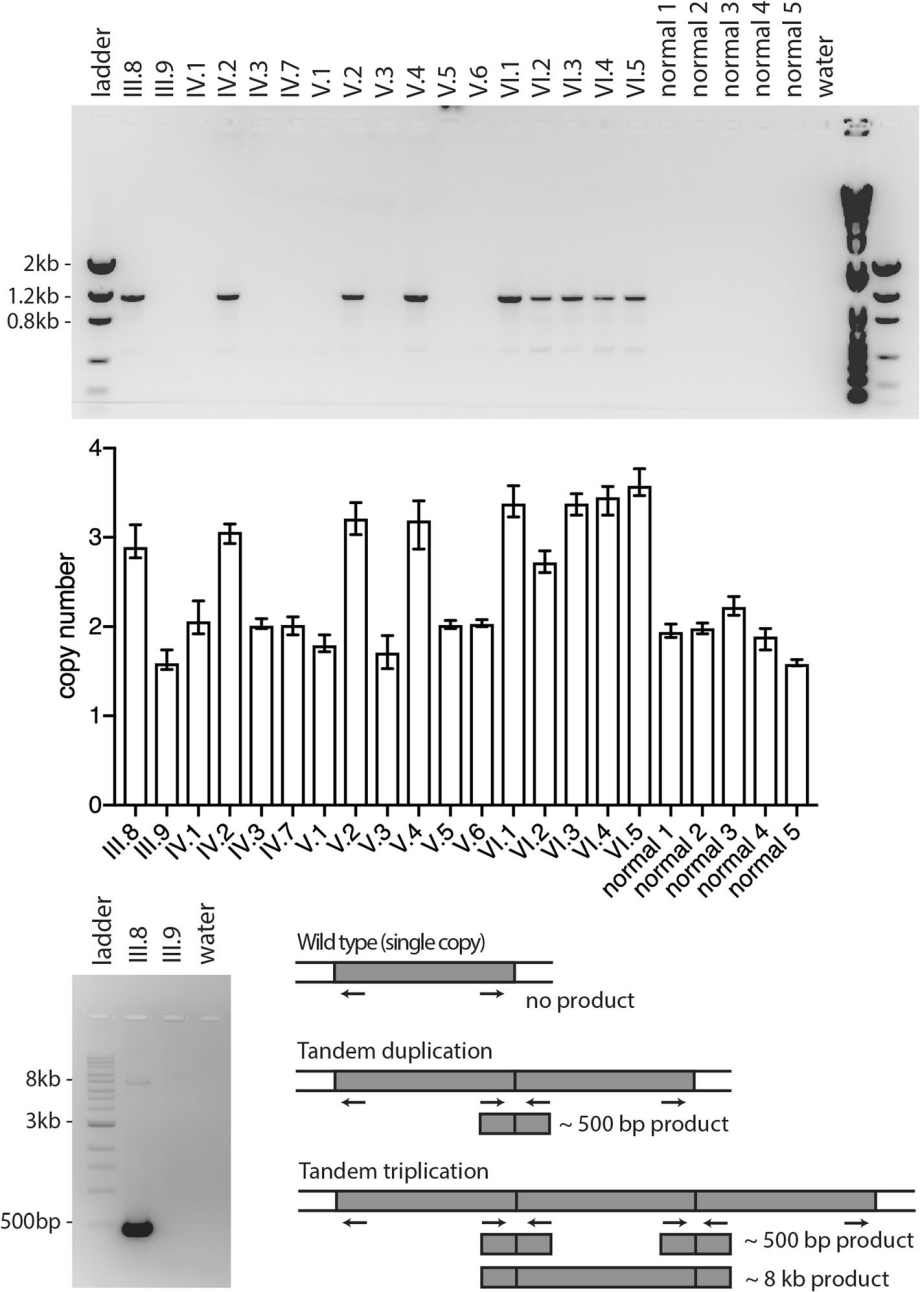
Genetic testing results. Top, 1% agarose gel with breakpoint polymerase chain reaction testing of cavitary optic disc anomaly pedigree and unrelated individuals. Middle, Results of pre-designed quantitative polymerase chain reaction copy number assay. Error bars represent minimum and maximum copy number as reported by CopyCaller software. Bottom left, 0.7% agarose gel electrophoresis of products of long range PCR. Bottom right, breakpoint primer design showing lack of product in wild type haplotype, ~ 500 bp product in tandem duplication, and ~ 500 bp product plus 8 kb product in tandem triplication

We tested 17 family members with breakpoint PCR (Fig. 3a) and detected the CNA in all four clinically affected family members (individuals III.8, IV.2, V.2, and V.4), as well as five younger family members in the sixth generation (individuals VI.1, VI.2, VI.3, VI.4, and VI.5). The clinically unaffected adults (of the third, fourth, and fifth generations) were negative for the CNA, as were five unrelated patients without ocular disease. The youngest member of the sixth generation (VI.6) was not tested due to age (4 years old).

#### Quantitative PCR assay

We further tested members in our CODA pedigree using a real time quantitative PCR copy number assay targeting the appropriately identified region within the CNA (hg19) chr12:56,244,285 (Fig. 3b). All unaffected adults and unrelated normal individuals analyzed had similar values, and this value was used to establish a baseline value for two copies during the analysis. The four affected adults and the five tested individuals in the sixth generation revealed a higher number of copies. Observed values for the number of copies in these individuals ranged from three to four. The results of this assay were affected by sample-specific error, the sources of which are unknown.

Family members of our CODA pedigree were further tested for the chromosome 12q CNA using a second quantitative PCR assay targeting nucleotides (hg19) chr12:56,241,440 that produced the same results (data not shown).

#### Long range PCR assay

To more precisely establish the number of copies present in the CNA, we performed long range PCR with a design similar to the breakpoint PCR assay. In the presence of a tandem triplication (i.e. four total copies including the normal allele), PCR amplification would generate an ~ 8 kb product consisting of the end of one copy, an entire second copy, and the beginning of a third copy (Fig. 3d). This product would occur in addition to the ~ 500 bp product consisting of the end of one copy and the beginning of the next. In the presence of a tandem duplication (i.e. three total copies), PCR amplification would generate only a ~ 500 bp product. We tested DNA from affected individual III.8 with this long range PCR assay and found both a ~ 500 bp product and a ~ 8 kb product (Fig. 3c). This is consistent with a tandem triplication, or four total copies of the region per genome (three copies on one chromosome and one copy on the other chromosome). Sanger sequencing confirmed that this product was of the expected identity (data not shown).

### Phenotype of clinically unaffected individuals

**Fig. 4:**
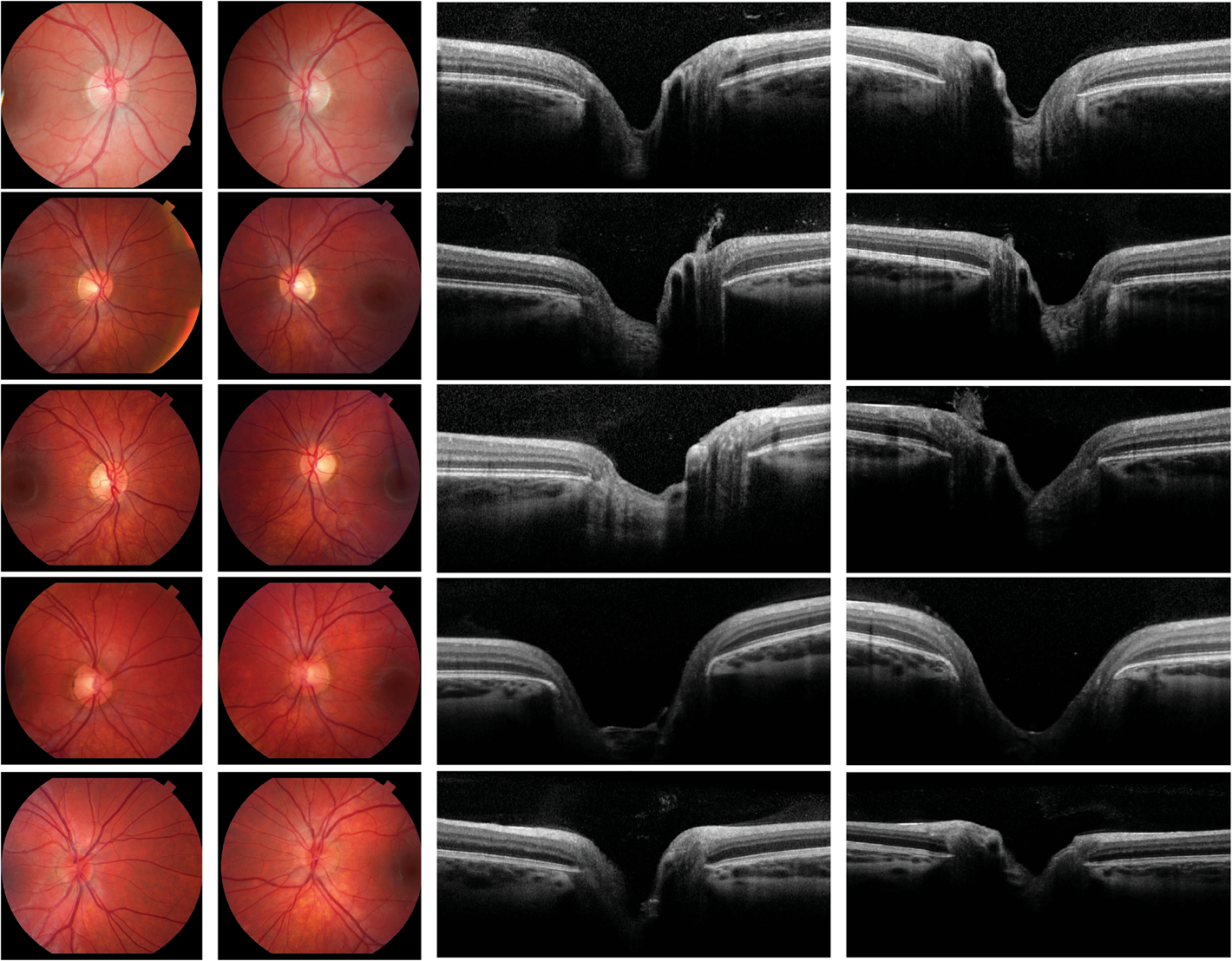
Optic nerve photographs and horizontal optical coherence tomography of the optic nerve. The first and third columns depict the right eye and second and fourth columns depicts the left eye of individuals in the sixth generation of the cavitary optic disc anomaly pedigree. Top row, individual VI.1. Second row, individual VI.2. Third row, individual VI.3. Fourth row, individual VI.4. Fifth row, individual VI.5

The unaffected adults and the children in the sixth generation were characterized by clinical exam. For further clinical characterization, stereoscopic optic nerve photos and optic nerve OCTs (Fig. 4) were independently reviewed by two additional ophthalmologists, a glaucoma specialist and a neuro-ophthalmologist. All phenotyping was performed prior to genetic analysis. There was no evidence of optic nerve abnormality in any of the five minors in the sixth generation, although they were all positive for the CNA.

### In silico analysis

**Fig. 5:**
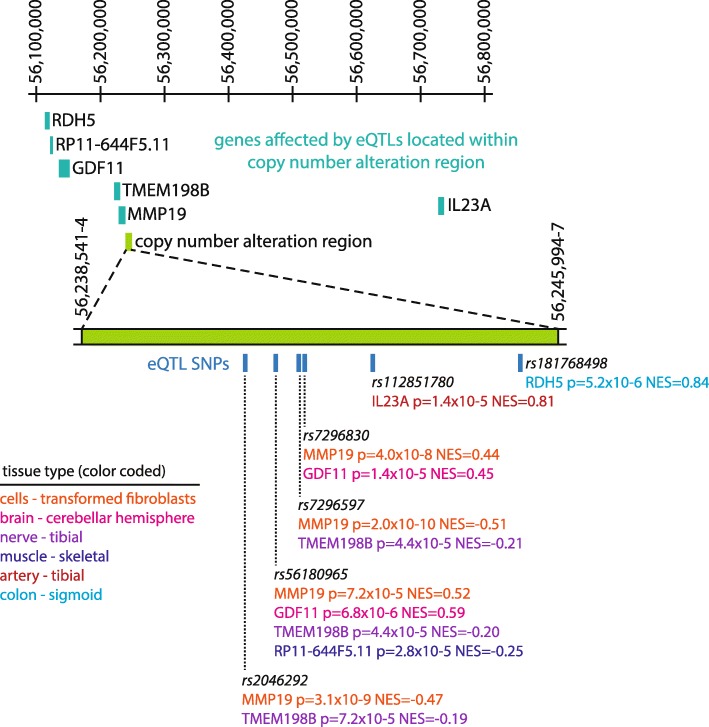
Locations of expression quantitative trait loci (eQTLs) within the copy number alteration region. Single nucleotide polymorphisms (SNPs) are mapped out within the copy number alteration region with color coding indicating the tissue type in which they are found to act as eQTLs

Using GTEx, we investigated the CNA region for the presence of known expression quantitative trait loci (eQTLs) (Fig. 5). Six single nucleotide polymorphisms (SNPs) within the CNA region were found to alter expression of at least four genes in *cis*. Some of the SNPs acted as eQTLs for multiple genes. Four eQTLs affecting *MMP19* expression were found in transformed fibroblasts. Two eQTLs affecting *GDF11* were found in the cerebellar hemisphere of the brain, and three eQTLs affecting *TMEM198B* were found in the tibial nerve. One eQTL affected *IL23A* in the tibial artery.

## Discussion

Optic nerve coloboma, morning glory, and optic pit are congenital optic disc abnormalities. The optic disc abnormalities in CODA were previously described as being congenital as well, although some affected individuals were reported to have progressive optic cup enlargement [17]. We provide evidence that optic disc abnormalities in CODA sometimes appear in adulthood. The development of optic disc abnormality later in life could explain why maculopathy is adult-onset in these individuals. Three of the four family members with CODA in our pedigree (individuals IV.2, V.2, and V.4) had progressive optic disc changes that were observed over 2 to 9 years. One individual presented with a normal left optic nerve, but subsequently developed an optic pit with macular fluid after nine years. Two other individuals in the pedigree presented with optic pits in both eyes and showed increasing optic cup enlargement over time in the absence of surgery, laser, or elevated intraocular pressure.

Progression from a normal optic nerve to an optic pit outside of the context of glaucoma has been described previously by Perkins et al. in two unrelated patients who presented with macular schisis and normal-appearing optic nerves who subsequently showed optic nerve head cavitation when fluid spontaneously resolved several months later [18]. These reports could represent a different phenomenon from the family described here, in which the optic pit preceded optic nerve cupping and macular fluid development. The late stage phenotype of severely enlarged cup and maculopathy is reminiscent of previous descriptions of macular schisis and subretinal fluid associated with glaucomatous optic nerve cupping [19], although most of these patients had high intraocular pressure, unlike the patients in this family.

The most notable finding in this pedigree was that all five of the children with ages ranging from 4 to 17 years were positive for the CNA by two independent assays, yet demonstrated normal optic nerves by clinical exam and OCT. One possible explanation for this could be incomplete penetrance. However, there was complete penetrance in all tested adults in this pedigree and only one asymptomatic carrier in published pedigrees including dozens of individuals [1–3, 20]. Another possible explanation could be that the CNA locus is not associated with the disease, but this explanation is not viable given the excellent segregation of the CNA with disease in the 12 adults in this pedigree as well as an independent family [7]. The more likely explanation is that the children positive for the CNA are at high risk for developing CODA. As illustrated by patient V.2, a normal optic nerve may later develop a pit, followed by severe optic nerve cupping. Although there is currently no way to prevent macular fluid from developing, early detection and treatment of visually significant macular fluid may improve prognosis. Therefore, we recommend baseline and regular monitoring in patients with a first degree relative with hereditary optic pits or atypical colobomas, especially if they have the CNA described in this publication.

The optimal method to test for this specific CNA is to perform the simple breakpoint PCR assay we developed that does not require next generation sequencing or quantitative PCR. Previously, the CNA boundaries were defined by array comparative genomic hybridization, but we were able to define the boundaries precisely by Sanger sequencing and demonstrate that a simple breakpoint PCR assay can now be used to check for the presence of this disease-associated CNA in autosomal dominant CODA families. We also establish that the triplication is tandem and non-inverted.

Although the triplication is constant and segregates with disease through the generations, variable expressivity is a cardinal feature of autosomal dominant CODA. We describe a four generational pedigree, which like other pedigrees described in the literature [1–3, 20], shows varied optic nerve morphology, unilateral and bilateral expression of optic nerve and macular disease, and variable age of onset of maculopathy. Changes in optic nerve appearance over time may contribute to the variable phenotypes observed in different individuals with CODA in cross-sectional studies. In silico analysis revealed that eQTLs identified in the CNA region alter expression of four genes, including *MMP19.* Variation in one or more of these genes along with the CNA may also explain the observed variable expressivity. Very few disorders of Mendelian inheritance are associated with intergenic CNAs, and of these, homozygous deletions predominate, most likely due to the lack of sensitivity of next generation sequencing for duplications, triplications, and heterozygous deletions [21]. Once an intergenic CNA is associated with a disease, establishing disease causality is a significant challenge, unlike loss of function or gain of function mutations in genes.

The CNA described here is upstream of and may act as an enhancer for the *MMP19* gene, which encodes matrix metalloproteinase 19, an enzyme that degrades extracellular matrix and is expressed in the optic nerve head [22]. Through a query of GTEx, a database of eQTLs [16], we identified 6 SNPs in the CNA that likely alter gene expression of four genes and two pseudogenes by 12 cis eQTLs. eQTLs affecting expression of *MMP19* were found in transformed fibroblasts, and we identified other genes whose expression could be affected by this CNA, and therefore may play a role in the pathogenesis of optic pit in this family. In particular, SNPs within the CNA were associated with expression of *GDF11* in the brain, and *GDF11* encodes a TGF-beta ligand and affects the number of retinal ganglion cells and optic nerve diameter in mouse models [23]. Other genes identified by eQTL analysis include *IL23*, a cytokine, and *RDH5*, which encodes a retinol dehydrogenase of the visual cycle that is mutated in fundus albipunctatus. eQTLs can be significantly tissue-dependent [24]. We hypothesize that while gene expression in the brain may closely relate to expression in the optic nerve, non-eQTL tissue-specific expression assays could be employed in the future to assess the functional impact of intergenic CNAs [21].

A diverse regulatory role of the triplicated region could explain variable expressivity in CODA. The triplicated region could regulate expression of multiple nearby genes as identified by eQTL analysis. Thus, the age of onset of optic nerve or macular disease may depend on a complex interplay of the triplicated intergenic region with a delicate regulatory network that maintains the optic nerve cup over normal aging, but which when disrupted results in progressive excavation of a formerly normal optic nerve.

We observed that although the CNA localizes to an autosomal chromosome, there are more affected females than males in our pedigree as well as the other well characterized CODA pedigrees described to date [1, 3, 20]. Females comprise 66% of the 53 affected individuals described in four CODA pedigrees. This may be due to an abnormal secondary sex ratio rather than sex-specific penetrance, since females comprise 70% of the 71 offspring of affected females in these pedigrees. *MMP19* and *GDF11* are expressed in the endometrium [14], and although our CNA was not found to be eQTLs for these genes specifically in the uterus, they could potentially affect secondary sex ratio by mediating fertilization, implantation or embryonic death. Different intrauterine conditions are hypothesized to explain the abnormal sex ratio in offspring of carriers of the gene responsible for x-linked retinoschisis [25].

## Conclusions

Our evidence that some optic nerve anomalies are not congenital provides hope that a therapy could be designed to prevent optic nerve excavation and thereby prevent vision loss from maculopathy. In the cases we described, cupping of the optic nerve preceded macular fluid development. Although maculopathy can be treated by vitrectomy, laser, and gas, vision loss may still occur due to persistent fluid or due to laser of the papillomacular bundle. In this pedigree, only one out of the four affected individuals was not affected by permanent vision loss from this disease, emphasizing the need for periodic ocular examinations in asymptomatic family members. The CNA region associated with this phenotype regulates expression of up to four genes through *cis*-expression quantitative trait loci (cis-EQTLs), which may explain variable expressivity in this disease. Investigating how the targeted genes are mechanistically regulated by the chromosomal 12 intragenic triplication may lead to novel treatments.

## Additional file

Additional file 1: **Figure S1.** Spectral domain optical coherence tomography horizontal line scan through left optic nerve of affected individuals. From left to right, individuals III.8, IV.2, V.2, and V.4. (JPG 411 kb)

## Abbreviations

cis-EQTLs: *cis*-expression quantitative trait loci
CNA: Copy number alteration
CODA: Cavitary optic disc anomaly
DNA: Deoxyribonucleic acid
OCT: Optical coherence tomography
PCR: Polymerase chain reaction
SNP: Single nucleotide polymorphism
