# Supplementary figures and images for: Progressive optic nerve changes in cavitary optic disc anomaly: integration of copy number alteration and cis-expression quantitative trait loci to assess disease etiology
Source: BMC Med Genet. 2019 Apr 27;20:63. doi: 10.1186/s12881-019-0800-4 (PMC6487068; doi:10.1186/s12881-019-0800-4)

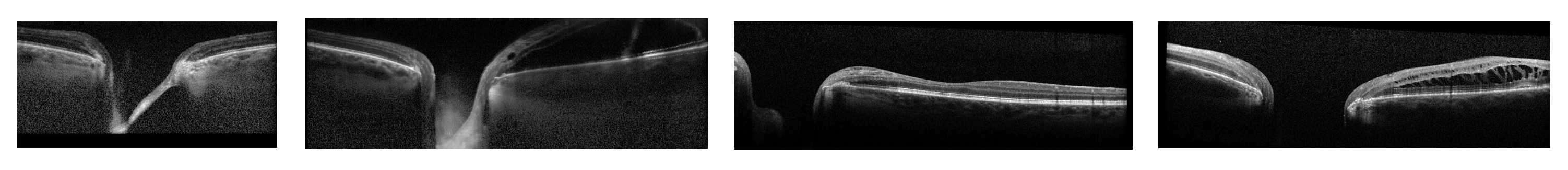

Supplement: Supplementary file 1 — Figure S1. Spectral domain optical coherence tomography horizontal line scan through left optic nerve of affected individuals. From left to right, individuals III.8, IV.2, V.2, and V.4. (JPG 411 kb) [file 12881_2019_800_MOESM1_ESM.jpg]
